# The Role of Endothelial-to-Mesenchymal Transition in Cardiovascular Disease

**DOI:** 10.3390/cells11111834

**Published:** 2022-06-03

**Authors:** Qianman Peng, Dan Shan, Kui Cui, Kathryn Li, Bo Zhu, Hao Wu, Beibei Wang, Scott Wong, Vikram Norton, Yunzhou Dong, Yao Wei Lu, Changcheng Zhou, Hong Chen

**Affiliations:** 1 Vascular Biology Program, Department of Surgery, Boston Children’s Hospital, Harvard Medical School, Boston, MA 02115, USA; qianman.peng@childrens.harvard.edu (Q.P.); dan.shan@childrens.harvard.edu (D.S.); kui.cui@childrens.harvard.edu (K.C.); kathryn.s.li@gmail.com (K.L.); bo.zhu@childrens.harvard.edu (B.Z.); hao.wu3@childrens.harvard.edu (H.W.); beibei.wang@childrens.harvard.edu (B.W.); scott.wong@childrens.harvrad.edu (S.W.); vikram.norton@childrens.harvard.edu (V.N.); yunzhou.dong@childrens.harvard.edu (Y.D.); yaowei.lu@childrens.harvard.edu (Y.W.L.); 2 Division of Biomedical Sciences, School of Medicine, University of California, Riverside, CA 92521, USA; changcheng.zhou@medsch.ucr.edu

**Keywords:** endothelial-to-mesenchymal transition, cell signaling, multidisciplinary and novel approaches, cardiovascular disease

## Abstract

Endothelial-to-mesenchymal transition (EndoMT) is the process of endothelial cells progressively losing endothelial-specific markers and gaining mesenchymal phenotypes. In the normal physiological condition, EndoMT plays a fundamental role in forming the cardiac valves of the developing heart. However, EndoMT contributes to the development of various cardiovascular diseases (CVD), such as atherosclerosis, valve diseases, fibrosis, and pulmonary arterial hypertension (PAH). Therefore, a deeper understanding of the cellular and molecular mechanisms underlying EndoMT in CVD should provide urgently needed insights into reversing this condition. This review summarizes a 30-year span of relevant literature, delineating the EndoMT process in particular, key signaling pathways, and the underlying regulatory networks involved in CVD.

## 1. Introduction

Endothelial cells (ECs) and mesenchymal cells are two distinct cell lineages that are both derived from the mesoderm. ECs are a heterogeneous cell population that exhibits tissue-specific properties [1,2]. Through adherens and tight junctions, ECs lining veins and arteries act as a barrier between the vessel wall and circulating blood, and the ECs lining the brain form the blood–brain barrier, whereas highly fenestrated pancreatic islet ECs allow for the release of molecules such as insulin from β cells into the bloodstream to modulate blood glucose levels [3,4,5,6]. ECs can be distinguished by the expression of cell–cell adhesion molecules, including platelet/EC adhesion molecule-1 (CD31/PECAM-1), vascular endothelial (VE)-cadherin, von Willebrand factor (vWF), tyrosine kinase with immunoglobulin-like and epidermal growth factor (EGF)-like domains 1 (TIE1), and TIE2 [7,8,9,10,11,12,13,14,15,16].

In contrast to endothelial cells, mesenchymal cells lack adherents and tight junctions, instead possessing a spindle or stellate shape that allows cells to freely move across the extracellular matrix and form the connective tissue that plays an important role in organ functions [3,17]. Mesenchymal cells, commonly referred to as mesenchymal stem cells (MSCs), have been reported to have the ability to differentiate into chondrocytes, osteocytes, and adipocytes [18,19,20,21,22,23], which express mesenchyme-specific markers, such as N-cadherin, α-smooth muscle actin (α-SMA), vimentin, fibroblast specific protein-1 (FSP-1, also known as S100A4), fibronectin, and smooth muscle protein 22α (SM22α) [7,8,24,25,26]. The contribution of mesenchymal cells to the pool of myofibroblasts or fibroblasts implicated in fibrotic disorders has been comprehensively documented in a variety of tissues [27,28,29,30,31].

The term “endothelial-to-mesenchymal transition” (EndoMT) is defined as the process through which endothelial cells differentiate into mesenchymal cells [23]. During heart development, endocardial ECs are the primary source of coronary vascular ECs, which, through EndoMT, produce mesenchymal cells featuring plastic and migratory properties [32]. In the normal physiological condition, this cell fate conversion is necessary to properly form the cardiac valves of the developing heart [33]. However, EndoMT recurs postnatally during the development of various cardiovascular diseases (CVDs), such as atherosclerosis, adult valve diseases, myocardial fibrosis, and pulmonary arterial hypertension (PAH) [34,35,36,37,38,39,40,41,42,43,44]. In the EndoMT transitional process, endothelial cells progressively lose endothelial-specific markers and gain mesenchymal phenotypes [45]. The expression of cell–cell adhesion proteins is downregulated, but mesenchyme-specific factors are increased [46]. It is worth noting that multiple signaling pathways that modulate EndoMT, such as bone morphogenetic protein (BMP)–transforming growth factor (TGFβ), vascular endothelial growth factor A (VEGFA), epidermal growth factor receptor, FGF, Notch, EGFR, PDGF [47,48,49,50,51,52,53,54,55], Wnt/β-catenin signaling, calcineurin–NFAT, and transcription factor GATA4-mediated transcriptional regulation, are involved in cardiovascular diseases [51,56]. Thus, manipulating EndoMT or its reversed process, mesenchymal-to-endothelial transition, may provide hitherto unprecedented therapeutic potentials.

This review summarizes the main cell signaling transduction pathway in EndoMT and EndoMT-mediated pathogenesis. Most investigations have been limited to exploring endothelial and mesenchymal cell markers in response to inducers for EndoMT; however, the molecular mechanisms regulating pathological EndoMT remain elusive. Thus, we focus on the role of TGFβ, PDGF, Wnt/β-catenin, and FGF signaling pathways regulating EndoMT. We note that the precise transduction may differ between cell types as some features might be tissue- or organ-dependent. We also emphasize the therapeutic target and preclinical application of the EndoMT for cardiovascular disease treatment.

## 2. Signaling Pathways Involved in the Regulation of EndoMT

### 2.1. TGFβ (Transforming Growth Factor-β)

**Figure 1:**
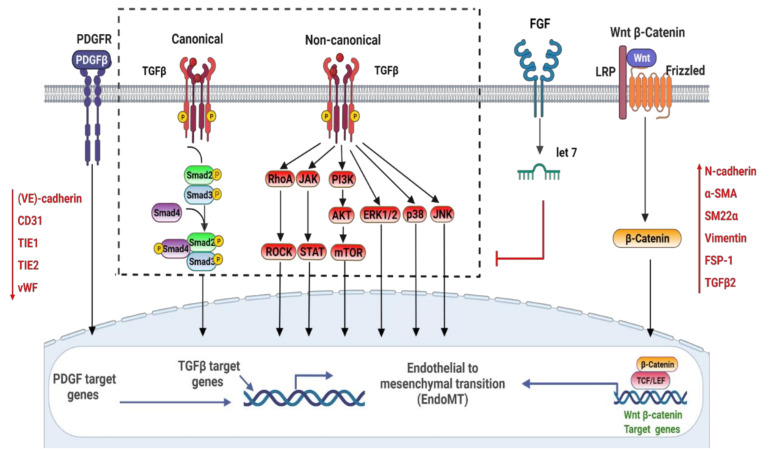
Signaling pathways involved in the regulation of EndoMT.

Currently, TGFβ signaling is the most well-investigated pathway recognized to induce EndoMT. Three mammalian isoforms of TGFβ (TGF-β1, TGF-β2, and TGF-β3) have been characterized, since TGF-β1 was initially identified in the early 1980s [57,58]. All three mammalian isoforms of TGFβ can induce EndoMT, with TGF-β2 playing a prominent role in doing so; additionally, different isoform- and species-specific functions have been identified in this process [59]. TGFβ binds to the tetrameric complex on the plasma membrane, which consists of two TGFβRI and two TGFβRII [60,61]. Both kinases possess dual specificity. Activin receptor-like kinases 1 and 5 (ALK1 and ALK5 receptors) are the prominent type I receptors in endothelial cells. ALK1 is activated by BMP9/10 (bone morphogenetic protein 9/10) and commonly leads to endothelial quiescence, while TGFβ induces ALK5 [62]. Once type I receptors are activated, the signal is transmitted from the cell membrane to the nucleus through the phosphorylation of a class of intracellular transcriptional effector proteins called mothers against decapentaplegic, also commonly known as Smads and SMA homologs [63]. Smad proteins are categorized into three categories: common Smads (coSmads, also known as Smad4 in vertebrates), receptor-associated Smads (R-Smads, Smad1/2/3/5/8), and inhibitory Smads (I-Smads, Smad6/7) [54,64,65,66,67]. TGFβ family ligands activate particular R-Smads via distinct receptor complexes [64,68], which can translocate into the nucleus and modulate certain transcriptional genes’ responses [69]. TGF-family members can also transduce signals via non-Smad pathways, such as Rho-like GTPase, the extracellular signal-regulated kinase MAP kinase (MAPK), and phosphatidylinositol3-kinase (PI3K)/AKT [70,71]. The challenge moving forward is to illustrate the complex mechanisms of TGFβ signaling with cross-talk to the other various signaling pathways and discover effective therapeutic agents targeting the TGFβ pathway in CVDs. Figure 1 presents the signaling pathways thought to be involved in EndoMT signal transduction.

### 2.2. PDGF

Platelet-derived growth factor (PDGF) signaling is important in cardiac development and has the ability to induce EndoMT. In mammals, a total of nine different genes encode four distinct PDGF chains (PDGF-A, PDGF-B, PDGF-C, and PDGF-D) [44,72,73], and all PDGFs are dimers of disulfide-linked polypeptide chains. PDGFs act via two receptor tyrosine kinases (RTKs) named PDGFR-α and PDGFR-β. Ligand binding induces the dimerization of the receptors, which is followed by activation through autophosphorylation [74]. The phosphorylated PDGF receptors cause the activation of downstream signaling pathways, Ras/mitogen-activating protein (MAP) kinase, including phospholipase C gamma (PLCγ) pathways and the phosphoinositide-3-kinase (PI3K)/AKT pathway, the proto-oncogene tyrosine kinase Src, members of the STAT family, and the tyrosine phosphatase SHP2 [44,75,76,77,78,79]. The expression of PDGF isoforms and PDGF receptors is enhanced during TGFβ-induced EndoMT. Exogenous PDGF-AA and PDGF-BB cooperate with the endogenous PDGF-A, or PDGF-B stimulated by TGF-β1, to synergistically induce EndoMT [73,80,81]. In addition, PDGF-AB selectively upregulates transcription factor Snail expression under hypoxia in human cardiac ECs [42].

### 2.3. Wnt/β-Catenin

Wnt/β-catenin signaling can act as a cofactor for TGFβ signaling. When the canonical Wnt pathway is inactive, β-catenin is maintained at low cytosolic levels by constitutive ubiquitination and proteasomal degradation. Additionally, β-catenin can interact with vascular endothelial cadherin at the cytoplasmic face of adherens junctions in non-Wnt–stimulated endothelial cells [82]. Wnt ligands interact with Frizzled receptors on the plasma membrane, altering intracellular catenin levels, the main effector of canonical Wnt signaling [83]. When the Wnt signaling pathway is activated, the phosphorylation of β-catenin is inhibited, and thus free β-catenin accumulates and translocates into the nucleus. In the nucleus, it enhances transcription of the lymphocyte enhancer factor/T-cell transcription factor (Lef/TCF) [84]. Wnt and TGF signaling may converge in the nucleus, where β-catenin interacts with Lef/TCF and Smad transcription factors to coordinate transcriptional control of shared target genes [84]. Wnt signaling is essential for the occurrence of EndoMT in endocardial cushions in the developing heart. Indeed, when there is a lack of β-catenin in cushion explants or endothelial cells, they fail to undergo EndoMT [85].

### 2.4. FGF

In mammals, the FGFR family is composed of four members (FGFR1–FGFR4). FGF ligand binding promotes FGFRs to dimerize and initiates trans-phosphorylate specific tyrosine residues in its cytoplasmic kinase domains, thereby causing FGFR activation. Subsequent phosphorylation takes place in the receptors’ cytoplasmic domains. Meanwhile, the constitutively docked fibroblast growth factor receptor substrate 2 alpha (FRS2α), an adaptor protein, establishes docking sites for certain cytoplasmic proteins. This, in turn, leads to the activation of downstream signaling cascades, including phospholipase C gamma (PLCγ) pathways, the phosphoinositide-3-kinase (PI3K)/AKT pathway, and Ras/mitogen-activating protein (MAP) kinase [50,86]. In addition to activating the aforementioned pathways, endothelial phenotype and function are also modulated by FGF signaling that counters TGFβ-driven EndoMT. In 1990, it was proven that FGF2 (fibroblast growth factor-2, also known as basic FGF) [50,87], a growth factor known to play critical roles in endothelial proliferation and vascular integrity, suppresses TGFβ signaling in endothelial cells [86,88]. In addition, recent mechanistic studies have revealed that FGF activation via endothelial FGFR1 suppresses TGFβ signaling and EndoMT. It was shown that activated FGFR1 recruits FRS2α, which induces let-7 microRNA expression and suppresses the expression of TGFβRI [89,90]. However, inflammatory cytokines, including TNF-α, IFN, and IL-1β, inhibit FGFR1 signaling and enhance EndoMT in some cultured endothelial cells [91].

## 3. EndoMT in In Vitro Studies

Extensive research has been conducted to elucidate the occurrence of EndoMT in in vitro studies. EndoMT is defined in these studies as cells that exhibit the following characteristics: (1) loss of endothelial properties, (2) coexpression of endothelial and mesenchymal markers, (3) increased migration, and (4) increased mesenchymal and myofibroblastic cells [23,91]. For example, Krenning et al. demonstrated that when human umbilical cord endothelial cells (HUVECs) were passaged and cultured with TGF-β1 and PDGF-BB, these cells lost their endothelial markers and developed spindly shapes while gaining the capacity to produce a variety of fibroblast-specific molecules. In one coagulation assay, HUVECs lost the function to prevent thrombin formation while acquiring a migratory capacity towards PDGF-BB signaling, gained contractile behavior similar to vascular smooth muscle cells, and produced smooth muscle protein 22α (SM22α) and α-SMA when cultured in mesenchymal differentiation medium. This research indicated that HUVECs could efficiently transdifferentiate into smooth muscle-like cells through endothelial-to-mesenchymal transdifferentiation [92]. Similar studies were conducted on cultured human coronary artery endothelial cells (HCAECs) and human aortic endothelial cells (HAECs) with TGF-β1 treatment [31,93,94,95]. In addition, overexpression of miR-200a was shown to block EndoMT in HAECs by inhibiting α-SMA, FSP-1, CD31, and VE-cadherin expression, regardless of the presence of TGF-β1 in human aortic endothelial cells [95]. More recently, it was reported that losartan, an angiotensin II type 1 receptor blocker, suppressed EndoMT in mitral valve endothelial cells by blocking the TGFβ-induced phosphorylation of the ERK pathway [96].

## 4. EndoMT in In Vivo Studies

EndoMT was initially described in transgenic mice models during experimentally induced cardiac fibrosis development via lineage tracing of endothelial and mesenchymal cells [97,98]. In one experimentally induced cardiac fibrosis study, it was underscored that TGFβ is crucial in mediating EndoMT. This EndoMT process is the main contributor to tissue fibrosis, acting as a profibrotic switch in cardiac fibrosis and other fibrotic diseases [31]. In other studies, macrophages were indicated to induce partial EndoMT. In turn, EndoMT regulates macrophage and endothelial cell phenotypes and lipid uptake, thereby affecting the surface structure and internal atherosclerotic plaque [99]. In addition, it has been demonstrated that miR-200c-3p/FERM2 is associated with EndoMT in human femoral arteries with atherosclerotic lesions [100]. Wnt2 protein has also been identified to express at a significantly high level in atherosclerotic lesions [101]. In 2001, Paranya et al. revealed the presence of transdifferentiation in vivo, with positive staining of mesenchymal cell marker α-SMA, and enhanced migration upon stimulation with PDGF-BB in a subpopulation of cells in frozen sections of aortic valves [102]. Recently, a study indicated that EndoMT is associated with alterations in the signaling of BMPR2, a gene that is mutated in 10% to 40% of cases of idiopathic PAH and in 70% of cases of familial PAH in rats [37,103]. In a recent study, Huang et al. generated an EC-specific PDGFR-β knockout transgenic mouse model and found a PDGF–NF-κB–HIF1-α–Snail axis that promotes VE-cadherin down-expression and activates mesenchymal-like transcriptional mechanisms and vessel abnormalities after myocardial infarction (MI) [104]. Table 1 summarizes the molecules involved, the functional changes seen related to EndoMT, the model system (in vitro/in vivo), and the cardiac disease studied.

## 5. Partial and Reversible EndoMT

ECs involve a progressive transition to mesenchymal via a fluid spectrum of intermediate cell states called partial EndoMT, which enables the temporary and reversible adoption of a hybrid endothelial-mesenchymal cell state [108]. This might may be triggered by signaling cross-talk regulatory mechanisms that limit complete progression through the EndoMT, preventing excessive mesenchymal transition. For example, FGF might antagonize TGFβ to restrict the complete EndoMT progression within the context of cardiovascular diseases [89]. However, the effects of the cross-talk and integrated signaling pathways should be evaluated at the level of the EndoMT master transcription factors. Snail and Slug inhibit the expression of one another, and both engage in distinct (as well as shared) signaling pathways that modulate partial and complete EndoMT [109]. Thus, activation of EndoMT counter pathways might limit the EndoMT at transcriptional levels and provide a novel strategy to reverse EndoMT-mediated CVDs. In addition, the time duration of chemical or physical stimuli may be another possible factor affecting the extent of EndoMT reversibility. In an experimental study, TGF-β1 pretreated ECs showed reversible EndoMT for culture times less than 10 days; however, ECs gained a stable mesenchymal phenotype and were irreversible when treated with TGF-β1 for 20 days [110].

## 6. EndoMT in Heart and Valve Development

During embryonic development, when endocardial cells differentiate into cardiomyocytes in the atrioventricular canal, they activate biosynthetic processes that contribute to the formation of the cardiac cushion mesenchyme and cardiac valves [36,111]. Initially, endocardial cells are delaminated from the endocardial sheet by transdifferentiating into mesenchymal cells and migrating into the cardiac jelly to form the cushion mesenchyme [112]. Then, the cushion progressively expands with accumulating mesenchymal cells primarily derived from endocardial cells and a portion of epicardial cells undergoing epicardial-to-mesenchymal transition [36]. The expansion of mesenchymal cells results in the elongation and remodeling of the valves, which lead to the formation of mature valve leaflets. In addition, lineage-tracing studies indicate that epicardial-derived mesenchymal descendants, which express PDGFRα or PDGFRβ, eventually give rise to pericytes and fibroblasts [36,113,114]. The EndoMT process has also been implicated in the embryonic development of multiple other vascular tissues, such as the formation of the abdominal aorta and the cardiac and semilunar valve [112]. Various stimuli, endothelin-1, angiotensin II, glucose, advanced glycation end-products, and inflammatory stimuli such as inflammatory mediators, growth factors, hypoxia, and proteases, can induce EndoMT via TGF-β signaling, which plays a vital role during the development of cardiovascular diseases [91,115,116,117,118,119,120].

## 7. EndoMT in Atherosclerosis

Atherosclerosis is a chronic inflammatory disease characterized by the formation of plaques in the intima, and endothelium is an important source for atherosclerotic plaque-associated mesenchymal cells from EndoMT [97,101,121,122]. Indeed, EndoMT has been shown in Cre–loxP-mediated genetic lineage tracing studies to play a vital role in the formation of the plaque deposits and in facilitating plaque instability leading to plaque rupture, which triggers the release of atherosclerotic nodules into the circulation [97,123]. In addition, endothelial-specific deletion of fibroblast growth factor receptor substrate 2 (FRS2) results in extensive EndoMT in the atherosclerotic plaque, which is accompanied by increased fibronectin deposition and neointima formation [105]. TGFβ signaling and transcription factor Snail were shown in response to shear stress, and the activated ECs initiated inflammatory responses via EndoMT in atherosclerosis [124]. Consistently, endothelial-specific TGFβRI/TGFβRII knockout in murine models of atherosclerosis has been shown to limit EndoMT, decrease inflammatory responses and plaque progression, and even enable plaque regression [31,85,125]. Moreover, patients with atherosclerosis have been significantly correlated with a high degree of endothelial TGFβ signaling and EndoMT activation [126,127,128]. These investigations provide mechanistic insights into the involvement of EndoMT in the progression of atherosclerosis, indicating that EndoMT acts as a link between inflammation and disturbed shear stress, with tissue remodeling promoting atherosclerotic plaque formation. All of this suggests that EndoMT could be a promising therapeutic target for preventing the development and progression of vulnerable plaques.

In recent years, single-cell RNA (scRNA) sequencing technology has facilitated the analysis of huge numbers of individual ECs in vascular tissue, revealing the complexity of atherosclerotic plaques in intricate detail. It is reported that transcriptional profiling of ECs from arterial tissue revealed cellular heterogeneity under disturbed flow [129,130]. For instance, mouse carotid arteries exposed to stable blood flow versus disturbed flow suggested that endothelial cells respond differently to stable versus disturbed flow at the genomic level with single-cell RNA sequencing analysis. Disturbed blood flow promoted the carotid arterial ECs into a wide variety of phenotypes from inflammatory to mesenchymal (i.e., EndoMT), immune cell-like, stem/progenitor-like, and hematopoietic phenotypes. Meanwhile, stable flow prevented, whereas the disturbed blood flow rapidly induced, robust atherosclerotic plaque development in the hypercholesterolemic mouse model [129]. This unbiased approach can characterize ECs in a complex arterial tissue without the prerequisite for sorting based on predefined markers.

## 8. EndoMT in Adult Valve Disease

Since EndoMT plays an essential role in the formation of heart valves, impairment of EndoMT can result in congenital valve disease. TGFβ is one of the four fundamental pathways (TGFβ, Notch, Wnt, and BMP) involved in valvulogenesis and also participates directly in impaired EndoMT in bicuspid and mitral prolapse valves, which are the most common congenital heart diseases [32,131,132]. Garside et al. identified that TGFβ signaling promotes EndoMT of endocardial cells and their invasion as mesenchymal cells into the cardiac cushions [53]. Also, Cre-mediated inactivation of TGFβRII in VE-cadherin-expressing ECs at E11.5 causes embryonic ventricular septal defect due to the failure of cushion fusion [31,85,133]. In addition, it has been reported that protein kinase R-like endoplasmic reticulum kinase (PERK) suppressed EndoMT in HUVECs under TGF-β1 stimulation in cardiac valve development. In healthy adult valves, interstitial valve cells are dormant fibroblasts. However, during disease progression, interstitial valve cells evolve into activated cells similar to myofibroblasts that express mesenchymal markers α-SMA [32,131], and subsequently differentiate into chondrocyte and osteoblast-like cells, which are characteristic of calcific aortic valve disease [134,135].

In a recent study, Bischoff et al. identified an unanticipated expression of CD45, one protein tyrosine phosphatase, in mitral valve endothelial cells post-MI in response to the stimulation of TGF-β1. In in vitro studies, they showed that ovine mitral VECs expressed a low basal level of endogenous CD45, which was increased significantly after being stimulated by TGF-β1. There were also concomitant increases in mesenchyme-specific factor α-SMA, additional EndoMT markers, TGF-β1, TGF-β3, collagen 1, and collagen 3, all of which were suppressed by the inclusion of one CD45 selective PTPase inhibitor. In vivo, CD45 expressed in the MV leaflet endothelium, accompanied by increasing VE-cadherin positive endothelial cells that express α-SMA and CD45, is significantly higher in inferior MI compared to in sham animals (adult sheep). This research suggested that CD45 promotes a maladaptive, profibrotic form of EndoMT in the mitral valve endothelium. It perhaps goes by post-translational processes such as the dephosphorylation of EndoMT-related molecules, which are linked to other valve illnesses such as calcific aortic valve disease (CAVD). This study, using clinically relevant large animal models, emphasized the complexity of the endothelium and indicated an unanticipated functional role for CD45 PTPase in EndoMT [136].

## 9. EndoMT in Myocardial Fibrosis

The critical role of EndoMT in the pathogenesis and progression of myocardial fibrosis has been described in recent years. For instance, endocardial cells can give rise to myofibroblasts through EndoMT after MI. These distinct changes accompany biochemical changes in cell morphology and polarity, featuring the decreased expression of endothelial markers, such as VE-cadherin, endothelial nitric oxide synthase (eNOS), CD31, and the acquisition of mesenchyme-specific factors, such as α-SMA, FSP-1, transgelin, and SM22a or calponin. Mechanistically, pSMAD2 and/or pSMAD3 were expressed in ECs in the injured heart, indicating that TGFβ signaling activation is involved in the embryonic EndoMT process. However, BMP7, a TGF-β1 antagonist, could significantly limit EndoMT-mediated EC transformation and the progression of cardiac fibrosis [137]. FGF activation causes a dramatic reduction in let-7 miRNA levels in tissue fibrosis that, in turn, upregulates the expression of TGFβ ligands and receptors, and activates TGFβ signaling in endothelial-to-mesenchymal transition [50,73,89,138]. Following myocardial infarction, partial EndoMT activation triggers robust new vessel generation [139]. Canonical Wnt/β-catenin pathway has also been shown to mediate EndoMT [83]. Lineage tracing utilizing Tcf21–Cre, Tbx18–Cre, Wt1–Cre, and Gata5–Cre lines indicated that endothelial-derived mesenchymal descendants that express PDGFRα or PDGFRβ subsequently give rise to pericytes and fibroblasts [111]. Moreover, the expression levels of EndoMT-related genes, including Twist, Snail, and Slug, are also significantly upregulated within the left ventricular myocardial tissues of individuals with end-stage cardiac failure. In addition to these signaling transduction pathways, microRNAs including miR-21, miRNA-24, or miR-29 have been implicated in regulating fibrosis after MI [140,141,142].

## 10. EndoMT in Pulmonary Arterial Hypertension

Pulmonary arterial hypertension is characterized by excessive pulmonary remodeling and intimal thickenings in the pulmonary arterial wall. Arciniegas et al. first suggested the role of EndoMT in the pathophysiology of chronic PAH, and further studies suggest that EndoMT has been implicated in primary PAH and PAH secondary to SSc [42]. In addition, Ranchoux et al. applied transmission electron microscopy, providing evidence that EndoMT is a key contributor to α-SMA positive cells in patients with primary pulmonary hypertension [143]. The histological assessment shows that α-SMA+/vWF+ endothelial cells are present in up to 5% of pulmonary vessels in patients with systemic sclerosis-associated pulmonary hypertension. The role of EndoMT in SSc-associated PAH pathology was investigated using a hypoxia/SU5416 mouse model. In addition, unambiguous expression of α-SMA indicated that EndoMT was involved in pulmonary arterial remodeling in intimal and plexiform lesions from PAH secondary to SSc lungs [144].

Moreover, proinflammatory mediators, such as tumor necrosis factor-α (TNF-α), IL-1β, IL-6, and IL-10, also induce EndoMT implicated in pulmonary hypertension [103,107]. Furthermore, several signaling pathways, such as TGFβ, Wnt/β-catenin, and the transcription factors Snail, Slug, and Twist1, are related to pulmonary hypertension involved in EndoMT [42,145,146]. These novel findings offer solid evidence for the role of EndoMT in both primary PAH and PAH secondary to SSc, which might provide a promising therapeutic target to inhibit and even reverse pulmonary vascular excessive remodeling for PAH [42,144].

## 11. Perspective

There has been a diverse interest in studying the role of EndoMT in cardiovascular disease; in fields such as systems biology, biophysics, stem cell biology, and pathology, research on EndoMT has been expanding rapidly in recent decades [97]. However, one major challenge is the translation of the current knowledge of EndoMT heterogeneity and plasticity into clinical practice. Preclinical studies of inhibitors majorly focus on fibrosis complications through EndoMT. For example, hepatocyte growth factor, losartan, scutellarin, BMP-7, and relaxin have been demonstrated to repress EndoMT and attenuate cardiac fibrosis [96,124,147,148,149,150]. These inhibitors of EndoMT could be therapeutic candidates for treating other diseases where EndoMT occurs and contributes to the pathogenesis. It should be noted that many of the experimental techniques currently utilized in the field still suffer from significant limitations. Indeed, markers of endothelial cells for endothelial lineage identification, such as CD31, are also expressed by other cell types, and thus using a single marker can lead to false-positive results [151]. Moreover, due to the lack of unified and unambiguous EndoMT read-outs on the basis of endothelial and mesenchymal characteristics, cross-comparison between different research remains challenging. With many important aspects of EndoMT remaining unexplored, using multidisciplinary and novel approaches—such as scRNA-seq, ATAC-seq, computational models, live imaging, multi-omics, bioinformatics analysis, and mathematical modeling—will help us better understand the etiology of EndoMT and provide support for the treatment of a myriad of diseases associated with EndoMT [103,104,129,148,152,153,154].

## 12. Conclusions

Compelling evidence has demonstrated that EndoMT is implicated in cardiac development and cardiovascular disease, including atherosclerosis, adult valve diseases, myocardial fibrosis, and pulmonary arterial hypertension. Thus, EndoMT may be a promising target for therapeutic intervention. However, only a few drug candidates that target EndoMT have been investigated for preclinical use. Innovative approaches such as single-cell RNA (scRNA)-sequencing technology will allow for detailed profiling of EndoMT. Limiting EndoMT by suppressing its inducible pathways or by promoting its counter pathways provides a novel paradigm to combat EndoMT-mediated CVDs. Inhibitors that suppress TGFβ signaling-induced EndoMT would be an excellent starting point to guide producing potentially new class drugs that combat EndoMT-mediated cardiovascular diseases, the leading cause of patient death worldwide.

## Figures and Tables

**Table 1:** EndoMT in in vitro and in vivo studies.

Molecules Involved	Functional Changes Seen Related to EndoMT	Model System (In Vitro/In Vivo)	Cardiac Disease Studied	References
TGFβ	Cells lost endothelial markers; developed spindly shapes; gained the capacity to produce a variety of fibroblast-specific molecules. Regulated cell phenotypes and lipid uptake and cell signaling, acted as a profibrotic switch in cardiac fibrosis diseases, thereby affecting the surface structure and internal atherosclerotic plaque.	In vitro: mitral valve endothelial cells; HUVECs; HCAECs; and HAECs. In vivo: frozen sections of aortic valves from mature sheep; in atherosclerotic plaque in the mouse model.	Atherosclerosis; adult valve disease; cardiac fibrosis; pulmonary arterial hypertension.	[23,31,91,92,93,95,96,105]
miR-200a overexpression; miR-200c-3p	miR-200a overexpression blocked EndoMT: inhibited α-SMA, FSP-1, CD31, and VE-cadherin expression. miRNA-200c-3p promoted EndoMT.	In vitro: HAECs; HUVECs. In vivo: In human femoral arteries with atherosclerotic lesions; in the mouse model.	Cardiac fibrosis; atherosclerosis.	[95,100]
ERK pathway↓	Losartan suppressed EndoMT by blocking the TGFβ-induced phosphorylation of the ERK pathway.	In vitro: mitral valve endothelial cells.	Myocardial fibrosis	[96,106]
TGFβ1 treatment: PDGF-BB signaling↑; SM22α ↑; α-SMA↑	Unable to prevent thrombin formation; acquired and enhanced the migratory capacity.	In vitro: HUVECs; HCAECs; HAECs. In vivo: frozen sections of aortic valves from mature sheep.	Adult valve disease	[92,93,95]
Wnt2↑	Expressed significantly high in atherosclerotic lesions.	In vivo: in atherosclerotic lesions in the mouse model.	Atherosclerosis	[101]
BMPR2	BMPR2 mutated gene was related to idiopathic PAH.	In vivo: familial PAH in rats.	Pulmonary arterial hypertension	[37,103,107]
PDGFR-β↑ VE-cadherin↓	The PDGF–NF-κB–HIF1-α–Snail axis promoted VE-cadherin down-expression.	In vivo: in the mouse model.	Myocardial infarction	[104]

↑ indicates upregulation in affected group;↓ indicates down regulation in affected group.

## Data Availability

Not applicable.
